# Methyl jasmonate abolishes the migration, invasion and angiogenesis of gastric cancer cells through down-regulation of matrix metalloproteinase 14

**DOI:** 10.1186/1471-2407-13-74

**Published:** 2013-02-10

**Authors:** Liduan Zheng, Dan Li, Xuan Xiang, Ling Tong, Meng Qi, Jiarui Pu, Kai Huang, Qiangsong Tong

**Affiliations:** 1 Department of Pathology, Union Hospital of Tongji Medical College, Huazhong University of Science and Technology, 430022, Wuhan, Hubei Province, People’s Republic of China; 2 Clinical Center of Human Genomic Research, Union Hospital of Tongji Medical College, Huazhong University of Science and Technology, 430022, Wuhan, Hubei Province, People’s Republic of China; 3 Department of Surgery, Union Hospital of Tongji Medical College, Huazhong University of Science and Technology, 430022, Wuhan, Hubei Province, People’s Republic of China; 4 Department of Cardiology, Union Hospital of Tongji Medical College, Huazhong University of Science and Technology, 430022, Wuhan, Hubei Province, People’s Republic of China

**Keywords:** Gastric cancer, Methyl jasmonate, Matrix metalloproteinase 14, Specificity protein 1

## Abstract

**Background:**

Recent evidence indicates that methyl jasmonate (MJ), a plant stress hormone, exhibits anti-cancer activity on human cancer cells. The aim of this study is to determine whether sub-cytotoxic MJ can abolish the migration, invasion and angiogenesis gastric cancer cells.

**Methods:**

Human gastric cancer cell lines SGC-7901 and MKN-45 were treated with diverse concentrations of MJ. Cell viability, proliferation, migration, invasion and angiogenesis capabilities of cancer cells were measured by MTT colorimetry, EdU incorporation, scratch assay, matrigel invasion assay, and tube formation assay. Gene expression was detected by western blot and real-time quantitative RT-PCR. Binding of transcription factor on gene promoter was detected by chromatin immunoprecipitation.

**Results:**

Sub-cytotoxic (0.05 to 0.2 mM) MJ attenuated the migration, invasion and angiogenesis, but not the cell viability or proliferation, of gastric cancer cells in a time- and dose-dependent manner, with down-regulation of matrix metalloproteinase 14 (MMP-14) and its downstream gene vascular endothelial growth factor. Restoration of MMP-14 expression rescued the SGC-7901 and MKN-45 cells from sub-cytotoxic MJ-inhibited migration, invasion and angiogenesis. In addition, sub-cytotoxic MJ decreased the specificity protein 1 (Sp1) expression and binding on MMP-14 promoter, while restoration of Sp1 expression rescued the cancer cells from sub-cytotoxic MJ-mediated defects in MMP-14 expression, migration, invasion and angiogenesis.

**Conclusions:**

Sub-cytotoxic MJ attenuates the MMP-14 expression via decreasing the Sp1 expression and binding on MMP-14 promoter, thus inhibiting the migration, invasion and angiogenesis of gastric cancer cells.

## Background

Gastric cancer is one of the most common cancers worldwide [1]. In spite of the improvement of surgical and multimodal therapy, invasion and metastasis of cancer cells remains the main cause of gastric cancer-related death, with a 5-year survival rate below 30% [2]. Chemotherapy is an appropriate option with the hope of prolonged survival for gastric cancer patients [3]. Currently, over sixty percent of the anti-cancer agents in use are derived from natural sources, including plants, marine organisms and micro-organisms [4]. Plant-derived compounds, such as vinblastine, vincristine, topotecan, irinotecan, etoposide and paclitaxel, have been an important source of clinically useful anti-cancer agents [5], which possess therapeutic effects against cancer cells by modulating cell cycle, proliferation, and viability [5]. Thus, novel anti-cancer plant-derived substances and treatment regimens are of interest and warrant to be developed.

Plant stress hormones are natural bioregulators in plant intracellular signaling and defense in response to injury or environmental stresses, such as ultraviolet radiation, osmotic shock and heat [6]. Among the plant hormones, salicylic acid and its derivative aspirin are extensively studied as potential anti-cancer therapeutics and chemopreventive agents [6,7]. The jasmonate family, a group of plant stress hormones consisting of *cis*-jasmone, jasmonic acid, and methyl jasmonate (MJ), are fatty acid-derived cyclopentanones that occur ubiquitously in the plant kingdom and regulate plant developmental processes and adaptation to environment [8]. In recent years, emerging evidence has shown the anti-cancer effects of naturally occurring jasmonates and their synthetic derivatives [9,10]. In general, MJ has been found to be superior to *cis*-jasmone and jasmonic acid in terms of cytotoxicity and induction of apoptosis in human cancer cells [11,12], suggesting that MJ is a promising agent for the development of cancer therapeutics.

Our previous studies have demonstrated that MJ exerts anti-tumor properties through down-regulating the expression of proliferating cell nuclear antigen, X-linked inhibitor of apoptosis protein, and survivin [12,13]. We have also shown that cell permeable seven-residue peptide of Smac significantly enhances the growth inhibition effects of MJ on prostate cancer cells [14]. However, the potential anti-cancer effects of MJ on gastric cancer and the underlying mechanisms still remain largely unknown. In addition, most of the current studies focus on the cytotoxicity of MJ on cancer cells, while the effects of sub-cytotoxic MJ on the invasion, metastasis and angiogenesis of cancer cells warrant further investigation. In this study, we demonstrate, for the first time, that sub-cytotoxic MJ suppresses the migration, invasion and angiogenesis of gastric cancer cells through attenuating the expression of matrix metalloproteinase 14 (MMP-14) via decreasing the specificity protein 1 (Sp1) expression and its binding on MMP-14 promoter.

## Methods

### Cell culture

Human gastric cancer cell lines SGC-7901 (moderately differentiated) and MKN-45 (poorly differentiated) were obtained from the Type Culture Collection of Chinese Academy of Sciences (Shanghai, China). Human endothelial cell line HUVEC (CRL-1730) was purchased from American Type Culture Collection (Rockville, MD). The cells were grown in RPMI1640 medium (Life Technologies, Inc., Gaithersburg, MD) supplemented with 10% fetal bovine serum (Life Technologies, Inc.), penicillin (100 U/ml) and streptomycin (100 μg/ml). Cells were maintained at 37°C in a humidified atmosphere of 5% CO_2_. MJ (Sigma, St Louis, MO) was prepared into stock solutions at a concentration of 1 mol/L in anhydrous dimethyl sulfoxide (Sigma), and stored at −20°C. Confluent monolayers of cells were incubated with different concentrations of MJ for 6, 12 and 24 hrs as indicated. The 50% inhibitory concentration (IC_50_) of 24 hr exposure, defined as the drug concentration resulting in 50% reduction of cell viability compared to solvent control, was determined by Bliss’s software (Bliss Co, CA).

### Patient tissue samples

Approval to conduct this study was obtained from the Institutional Review Board of Tongji Medical College (approval number: 2010-S003). Specimens of surgically resected primary gastric carcinoma were collected from twenty patients at the Department of Surgery, Union Hospital of Tongji Medical College, Huazhong University of Science and Technology in Wuhan, China. Their pathological diagnosis was proven by at least two pathologists. Adjacent gastric mucosa that contained no macroscopic tumor was also obtained, and the non-neoplastic areas were subsequently verified by microscopic histology to be free of tumor infiltration. Fresh gastric cancer and non-neoplastic tissues were collected and stored at −80°C until use.

### Measurement of cell viability

Cancer cells were cultured in 96-well plates at 5 × 10^3^ cells per well and treated with MJ or solvent. Cell viability was monitored by the 2-(4,5-dimethyltriazol-2-yl)-2,5-diphenyl tetrazolium bromide (MTT, Sigma) colorimetric assay [15]. All experiments were done with 6–8 wells per experiment and repeated at least three times.

### Cell proliferation assay

Cancer cells were cultured in 96-well plates at 5 × 10^3^ cells per well, treated with MJ or solvent, and exposed to 50 μmol/L of 5-ethynyl-20-deoxyuridine (EdU, Ribobio, China) for additional 4 hrs at 37°C. The cells were fixed with 4% formaldehyde for 15 min and treated with 0.5% Triton X-100 for 20 min at room temperature. After washing with phosphate buffered saline (PBS) for three times, the cells of each well were reacted with 100 μl of 1 × Apollo ® reaction cocktail for 30 min. Subsequently, the DNA contents of cells in each well were stained with 100 μl of Hoechst 33342 (5 μg/ml) for 30 min and visualized under a fluorescent microscope.

### Scratch migration assay

Cancer cells were cultured in 24-well plates, treated with MJ or solvent, and scraped with the fine end of 1-ml pipette tips (time 0). Plates were washed twice with PBS to remove detached cells, and incubated with the complete growth medium. Cell migration was photographed using 10 high-power fields, at 0 and 24 hrs post-induction of injury. Remodeling was measured as diminishing distance across the induced injury, normalized to the 0 hr control, and expressed as outgrowth (μm) [16].

### Matrigel invasion assay

The Boyden chamber technique (transwell analysis) was performed as previously described [16]. Cancer cells were treated with MJ or solvent. Homogeneous single cell suspensions (1 × 10^5^ cells/well) were added to the upper chambers, and allowed to invade for 24 hrs at 37°C in a CO_2_ incubator. Migrated cells were stained with 0.1% crystal violet for 10 min at room temperature and examined by light microscopy. Quantification of migrated cells was performed according to published criteria [17].

### Tube formation assay

Fifty microliters of growth factor-reduced matrigel were polymerized on 96-well plates. HUVECs were serum starved in RPMI1640 medium for 24 hrs, suspended in RPMI1640 medium preconditioned with MJ- or solvent-treated cancer cells, added to the matrigel-coated wells at the density of 5 × 10^4^ cells/well, and incubated at 37°C for 18 hrs. Quantification of anti-angiogenic activity was calculated by measuring the length of tube walls formed between discrete endothelial cells in each well relative to the solvent control [18].

### Over-expression or knockdown of MMP-14 and Sp1

Human MMP-14 cDNA (1749 bp) expression construct was established as previously described [16]. Human Sp1 cDNA (2358 bp) was amplified from cancer tissue and subcloned into the *Hind* III and *Xha* I restrictive sites of pcDNA3.1/Zeo(+) (Invitrogen) (Additional file 1: Table S1). To restore the MJ-induced down-regulation of MMP-14 or Sp1, cancer cells were transfected with the recombinant vector pcDNA3.1-MMP14 or pcDNA3.1-Sp1 for 72 hrs before administration of MJ or solvent. The 21-nucleotide small interfering RNA (siRNA) targeting the encoding region of MMP-14 was chemically synthesized (RiboBio Co. Ltd; Additional file 1: Table S1) and transfected with Genesilencer Transfection Reagent (Genlantis, San Diego, CA). The scramble siRNA (si-Scb) was applied as controls (Additional file 1: Table S1). To monitor the transfection efficiency, the cancer cells were co-transfected with pEGFP-N1 (Clontech, Mountair View, CA).

### Western blot

Tissue or cellular protein was extracted with 1 × cell lysis buffer (Promega, Madison, WI). Western blot was performed as previously described [16,19], with antibodies specific for matrix metalloproteinase 7 (MMP-7), matrix metalloproteinase 9 (MMP-9), MMP-14, vascular endothelial growth factor (VEGF), Sp1, and β-actin (Santa Cruz Biotechnology, Santa Cruz, CA). Enhanced chemiluminescence substrate kit (Amersham, Piscataway, NJ) was used for the detection of signals with autoradiography film (Amersham).

### Real-time quantitative RT-PCR

Total RNA was isolated with RNeasy Mini Kit (Qiagen Inc., Valencia, CA). The reverse transcription reactions were conducted with Transcriptor First Strand cDNA Synthesis Kit (Roche, Indianapolis, IN). The PCR primers for MMP-7, MMP-9, MMP-14, VEGF, Sp1 and β-actin were designed by Premier Primer 5.0 software (Additional file 2: Table S2). Real-time quantitative RT-PCR with SYBR Green PCR Master Mix (Applied Biosystems, Foster City, CA) was performed as previously described [16,19], using ABI Prism 7700 Sequence Detector (Applied Biosystems). The fluorescent signals were collected during extension phase, Ct values of the samples were calculated, and the transcript levels were analyzed by 2^-ΔΔCt^ method.

### Chromatin immunoprecipitation

Chromatin immunoprecipitation (ChIP) assay was performed according to the manufacture’s instructions of EZ-ChIP kit (Upstate Biotechnology, Temacula, CA) [19]. The PCR primers surrounding the MMP-14 transcription start site were previously described [20]. Real-time quantitative PCR (qPCR) with SYBR Green PCR Master Mix was performed using ABI Prism 7700 Sequence Detector. The amount of immunoprecipitated DNA was calculated in reference to a standard curve and normalized to input DNA.

### Statistical analysis

Unless otherwise stated, all data were shown as mean ± standard error of the mean (SEM). The SPSS 12.0 statistical software (SPSS Inc., Chicago, IL) was applied for statistical analysis. Pearson’s coefficient correlation was applied for analyzing the relationship between Sp1 expression and MMP-14 transcript levels. Difference of cancer cells was determined by *t* test or analysis of variance (ANOVA).

## Results

### Sub-cytotoxic MJ attenuated the migration, invasion and angiogenesis of gastric cancer cells

**Figure 1:**
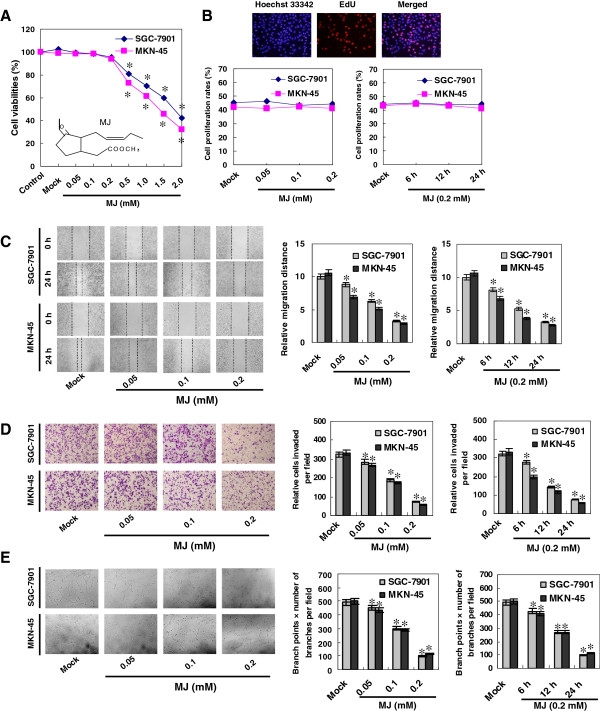
**Sub-cytotoxic MJ attenuated the migration, invasion and angiogenesis, but not the viabilities or proliferation, of gastric cancer cells.** Human gastric cancer cell lines SGC-7901 and MKN-45 were incubated with different concentrations of MJ as indicated. **A**, MTT colorimetric assay indicated that MJ suppressed the cell viabilities of gastric cancer cells with a range of concentrations (0.5 to 2.0 mM), while lower concentrations of MJ (0.05 to 0.2 mM) exerted no obvious cytotoxicity, when compared to those of solvent-treated (mock) cells. **B**, EdU incorporation assay revealed that sub-cytotoxic MJ (0.05, 0.1 and 0.2 mM) did not attenuate the proliferation of SGC-7901 and MKN-45 cells, when compared to that of mock cells. **C**, in scratch migration assay, administration of 0.05, 0.1 and 0.2 mM MJ attenuated the migration capabilities of SGC-7901 and MKN-45 cells in a dose- and time-dependent manner, when compared to those of mock cells. **D**, transwell analysis indicated that administration of sub-cytotoxic MJ (0.05, 0.1 and 0.2 mM) impaired the invasion capacities of SGC-7901 and MKN-45 cells in a dose- and time-dependent manner, than those of mock cells. **E**, the tube formation of endothelial cells was dose- and time-dependently suppressed by the medium preconditioned by treatment of gastric cancer cells with sub-cytotoxic MJ (0.05, 0.1 and 0.2 mM), than that of mock cells. The symbol (*) indicates a significant decrease from mock.

Since previous studies imply the anti-metastatic and anti-angiogenic properties of MJ [21,22], we hypothesized that MJ might influence the migration, invasion and angiogenesis of cultured gastric cancer cells. We first identified the sub-cytotoxic concentrations of MJ via a dose–response analysis in SGC-7901 and MKN-45 cells. As shown in Figure 1A, MJ suppressed the cell viabilities of gastric cancer cells with a range of concentrations (0.5 to 2.0 mM), while lower concentrations of MJ (0.05 to 0.2 mM) exerted no obvious cytotoxicity. The IC_50_ of MJ on SGC-7901 and MKN-45 cells were 1.72 and 1.24 mM, respectively. EdU incorporation assay was applied to further study the influence of sub-cytotoxic MJ on the proliferation of gastric cancer cells. As shown in Figure 1B and Additional file 3: Figure S1, administration of sub-cytotoxic (0.05, 0.1 and 0.2 mM) MJ did not attenuate the cell viabilities or proliferation. In scratch migration assay, sub-cytotoxic MJ attenuated the migration capabilities of SGC-7901 and MKN-45 cells in a dose- and time-dependent manner (Figure 1C). Transwell analysis showed that gastric cancer cells treated with sub-cytotoxic (0.05, 0.1 and 0.2 mM) MJ presented a dose- and time-dependently impaired invasion capacity than solvent-treated (mock) cells (Figure 1D). The tube formation of endothelial cells was dose- and time-dependently suppressed by the medium preconditioned by treatment of gastric cancer cells with 0.05, 0.1 and 0.2 mM MJ (Figure 1E). However, the proliferation of endothelial HUVEC cells was not affected by sub-cytotoxic MJ (Additional file 4: Figure S2), ruling out the possibility that sub-cytotoxic MJ affected the angiogenesis through direct cytotoxicity on endothelial cells. These results indicated that sub-cytotoxic MJ attenuated the migration, invasion and angiogenesis of gastric cancer cells *in vitro*.

### Sub-cytotoxic MJ down-regulated the expression of MMP-14, but not of MMP-7 and MMP-9, in gastric cancer cells

**Figure 2:**
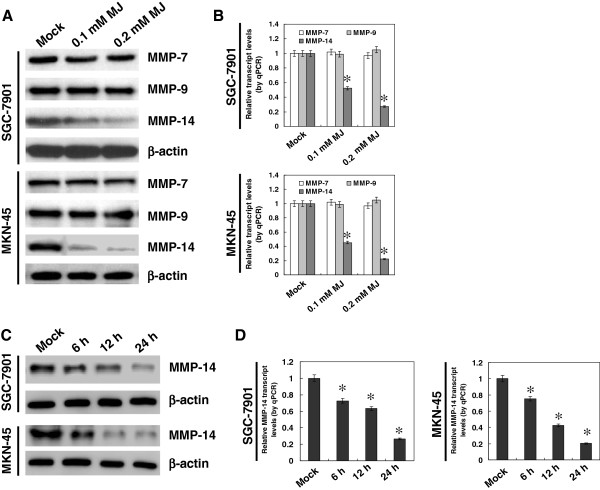
**Sub-cytotoxic MJ down-regulated the expression of MMP-14 in gastric cancer cells.** Human gastric cancer cell lines SGC-7901 and MKN-45 were incubated with sub-cytotoxic concentrations of MJ as indicated. **A** and **B**, western blot and real-time quantitative RT-PCR indicated that administration of sub-cytotoxic (0.1 and 0.2 mM) MJ to SGC-7901 and MKN-45 cells for 24 hrs resulted in a decrease in the expression of MMP-14, but not of MMP-7 or MMP-9, when compared to that of solvent-treated (mock) cells. **C** and **D**, western blot and real-time quantitative RT-PCR indicated that administration of 0.2 mM MJ to SGC-7901 and MKN-45 cells for 6, 12, and 24 hrs, resulted in the decrease of MMP-14 expression in a time-dependent manner, than that of mock cells. The symbol (*) indicates a significant decrease from mock.

Since previous studies reveal the critical roles of MMP-7, MMP-9 and MMP-14 in the invasion and metastasis of gastric cancer [23], we hypothesized that MJ might influence the expression of these genes. Western blot indicated that administration of sub-cytotoxic (0.1 and 0.2 mM) MJ resulted in a decrease in the expression of MMP-14, but not of MMP-7 or MMP-9, in cultured gastric cancer SGC-7901 and MKN-45 cells (Figure 2A). Real-time quantitative RT-PCR revealed the decreased transcript levels of MMP-14, but not of MMP-7 or MMP-9, in gastric cancer cells treated with sub-cytotoxic MJ (Figure 2B). In addition, sub-cytotoxic MJ-mediated inhibition on MMP-14 expression was in a time-dependent manner (Figure 2C and D). These results indicated that sub-cytotoxic MJ suppressed the MMP-14 expression in gastric cancer cells.

### Sub-cytotoxic MJ suppressed the expression of VEGF in gastric cancer cells

**Figure 3:**
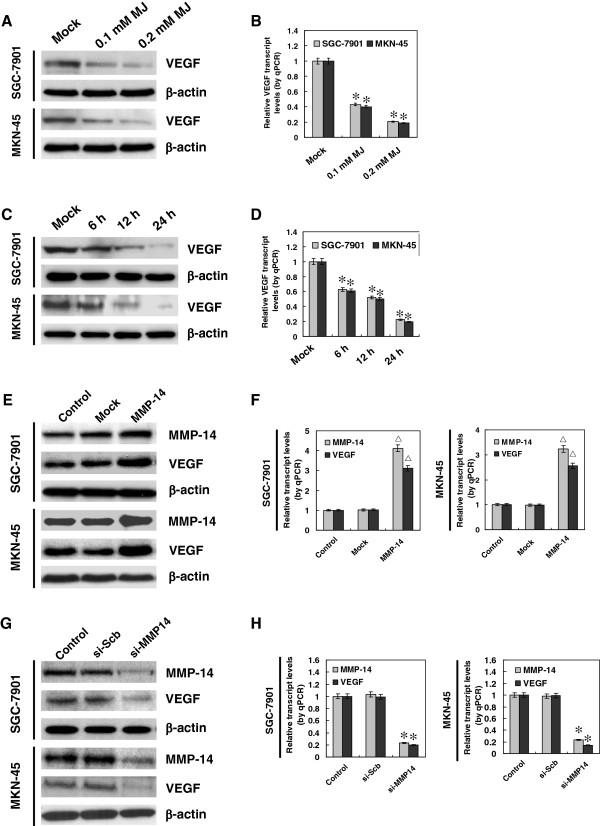
**Sub-cytotoxic MJ suppressed the expression of VEGF in gastric cancer cells.** Human gastric cancer cell lines SGC-7901 and MKN-45 were incubated with sub-cytotoxic concentrations of MJ as indicated. **A** and **B**, western blot and real-time quantitative RT-PCR indicated that administration of sub-cytotoxic (0.1 and 0.2 mM) MJ to SGC-7901 and MKN-45 cells for 24 hrs resulted in a decrease in the expression of VEGF, when compared to that of solvent-treated (mock) cells. **C** and **D**, western blot and real-time quantitative RT-PCR indicated that administration of 0.2 mM MJ to SGC-7901 and MKN-45 cells for 6, 12, and 24 hrs, resulted in the decrease of VEGF expression in a time-dependent manner, than that of mock cells. **E** and **F**, 72 hrs post-transfection of MMP-14 expression vector into SGC-7901 and MKN-45 cells, western blot and real-time quantitative RT-PCR indicated the over-expressed MMP-14 and VEGF than that of empty vector-transfected (mock) cells. **G** and **H**, 24 hrs post-transfection of si-MMP14 (100 nmol/L) into SGC-7901 and MKN-45 cells, western blot and real-time quantitative RT-PCR indicated the down-regulated MMP-14 and VEGF than those transfected with scramble siRNA (si-Scb, 100 nmol/L). The symbols (* and △) indicate a significant decrease and a significant increase from mock or si-Scb, respectively.

Since previous studies indicate that MMP-14 can regulate the VEGF expression in breast cancer cells [24], and combining the evidence that VEGF participates in the angiogenesis [25], we hypothesized that sub-cytotoxic MJ might also influence the expression of VEGF in gastric cancer cells. Western blot and real-time quantitative RT-PCR indicated that administration of sub-cytotoxic (0.1 and 0.2 mM) MJ resulted in a significant decrease of VEGF expression in gastric cancer SGC-7901 and MKN-45 cells, which was consistent with MMP-14 reduction (Figure 3A and B). In addition, sub-cytotoxic MJ-mediated inhibition on VEGF expression was in a time-dependent manner (Figure 3C and D). Furthermore, over-expression or knockdown of MMP-14 promoted or suppressed the VEGF expression in gastric cancer cells, respectively (Figure 3E, F, G and H), suggesting that as a direct downstream gene of MMP-14, the change of VEGF expression in sub-cytotoxic MJ-treated cancer cells may be due to the down-regulation of MMP-14.

### Over-expression of MMP-14 rescued sub-cytotoxic MJ-mediated suppression on VEGF expression, migration, invasion and angiogenesis of gastric cancer cells

**Figure 4:**
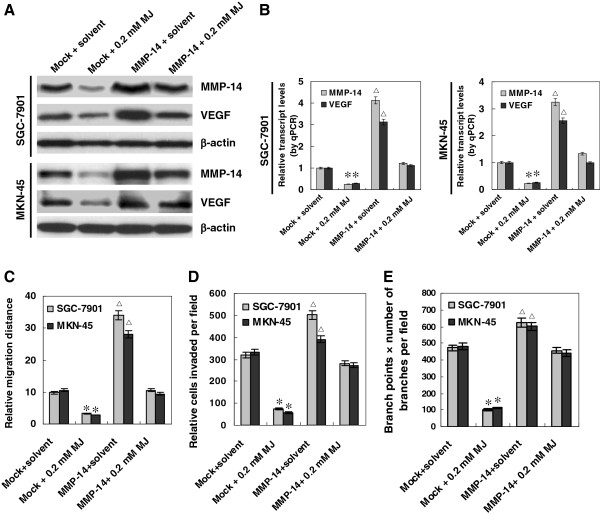
**Restoration of MMP-14 rescued sub-cytotoxic MJ-mediated suppression on VEGF expression, migration, invasion and angiogenesis of gastric cancer cells.** Human gastric cancer cell lines SGC-7901 and MKN-45 were transfected by MMP-14 expression vector for 72 hrs, and incubated with sub-cytotoxic MJ for 24 hrs. **A** and **B**, western blot and real-time quantitative RT-PCR indicated that transfection of SGC-7901 and MKN-45 cells with MMP-14 construct rescued the MJ-attenuated expression of MMP-14 and VEGF, when compared to those transfected with empty vector (mock) and treated with solvent. **C**, in scratch migration assay, over-expression of MMP-14 promoted the migration of SGC-7901 and MKN-45 cells, and rescued the 0.2 mM MJ-induced inhibition on the migration of cancer cells, when compared to that of solvent-treated mock cells. **D**, transwell analysis indicated that restoration of MMP-14 expression rescued the SGC-7901 and MKN-45 cells from 0.2 mM MJ-induced suppression of invasiveness, when compared to that of solvent-treated mock cells. **E**, restoration of MMP-14 expression in SGC-7901 and MKN-45 cells rescued the 0.2 mM MJ-induced suppression of angiogenesis, when compared to that of solvent-treated mock cells. The symbols (* and △) indicate a significant decrease and a significant increase from solvent-treated mock cells, respectively.

To further investigate the role of MMP-14 down-regulation in MJ-induced decrease in the migration, invasion and angiogenesis, MMP-14 expression construct was transfected into gastric cancer cells. The transfection efficiency was monitored by co-transfection with the enhanced green fluorescent protein (EGFP) reporter vector. Seventy-two hrs post-transfection, EGFP expressed within the cytoplasm of cancer cells, with the transfection efficiency around 60% (Additional file 5: Figure S3). As shown in Figure 4A and B, transfection of SGC-7901 and MKN-45 cells with MMP-14 construct restored the sub-cytotoxic MJ-attenuated expression of MMP-14 and VEGF. Restoration of MMP-14 expression rescued the SGC-7901 and MKN-45 cells from their defects in migration, invasion, and angiogenesis induced by sub-cytotoxic (0.2 mM) MJ (Figure 4C, D, and E). These results suggested that sub-cytotoxic MJ-induced suppression of migration, invasion and angiogenesis of gastric cancer cells, at least in part, was due to down-regulation of MMP-14 and its downstream gene VEGF.

### Sub-cytotoxic MJ suppressed the expression and binding of Sp1 on MMP-14 promoter

**Figure 5:**
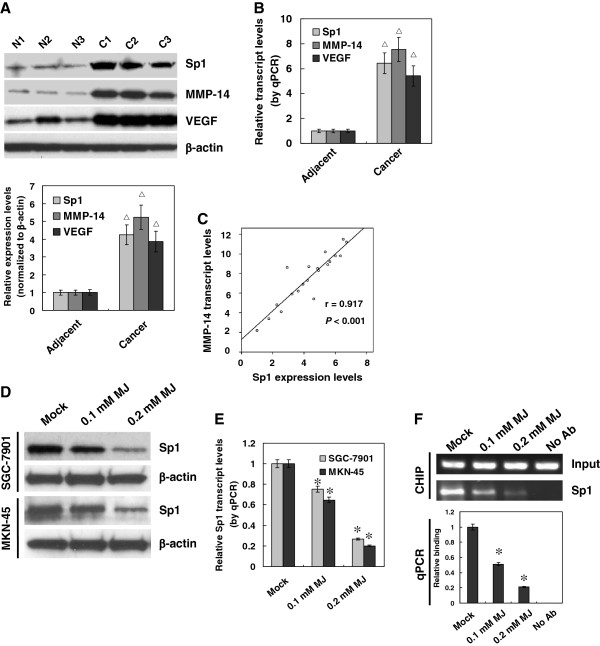
**Sub-cytotoxic MJ suppressed the Sp1 expression and binding on MMP-14 promoter in gastric cancer cells. A** and **B**, cancerous (**C**) and adjacent non-neoplastic (N) tissues from twenty gastric cancer patients were collected for the analysis of Sp1, MMP-14, and VEGF expression. Western blot and real-time quantitative RT-PCR indicated that the expression of Sp1, MMP-14, and VEGF was significantly higher in gastric cancer tissues than that of adjacent neoplastic tissues. **C**, Pearson’s coefficient correlation analysis demonstrated a positive correlation between Sp1 protein and MMP-14 transcript levels in gastric cancer tissues (r = 0.917, *P* < 0.001). **D** and **E**, western blot and real-time quantitative RT-PCR indicated that administration of 0.1 and 0.2 mM MJ to SGC-7901 and MKN-45 cells for 24 hrs, resulted in decreased Sp1 expression than that of solvent-treated (mock) cells. **F**, ChIP assay and real-time quantitative PCR indicated that administration of 0.1 and 0.2 mM MJ to SGC-7901 and MKN-45 cells for 24 hrs, resulted in decreased Sp1 binding on MMP-14 promoter than that of mock cells. There were no PCR products for “no-antibody” (No Ab) control. The symbols (* and △) indicate a significant decrease and a significant increase from adjacent tissues or mock, respectively.

Previous studies have revealed the critical role of transcription factor Sp1 in the regulation of MMP-14 expression in cancer cells [20]. To further explore the underlying mechanism for sub-cytotoxic MJ-induced MMP-14 down-regulation, cancerous and adjacent non-neoplastic tissues from twenty gastric cancer patients were collected for the analysis of Sp1, MMP-14, and VEGF expression. Western blot and real-time quantitative RT-PCR indicated that the expression of Sp1, MMP-14, and VEGF was significantly higher in gastric cancer tissues than that of adjacent non-neoplastic tissues (Figure 5A and B). Importantly, there was a positive correlation between Sp1 protein and MMP-14 transcript levels in gastric cancer tissues (Figure 5C). Administration of sub-cytotoxic (0.1 and 0.2 mM) MJ resulted in a decrease in the Sp1 expression in gastric cancer SGC-7901 and MKN-45 cells (Figure 5D and E). ChIP assay further revealed the decreased binding of Sp1 on MMP-14 promoter in cancer cells treated with sub-cytotoxic MJ (Figure 5F). These findings indicated that sub-cytotoxic MJ attenuated the MMP-14 expression via decreasing the Sp1 expression and binding on MMP-14 promoter in gastric cancer cells.

### Restoration of Sp1 rescued sub-cytotoxic MJ-mediated suppression on MMP-14 expression, migration, invasion and angiogenesis of gastric cancer cells

**Figure 6:**
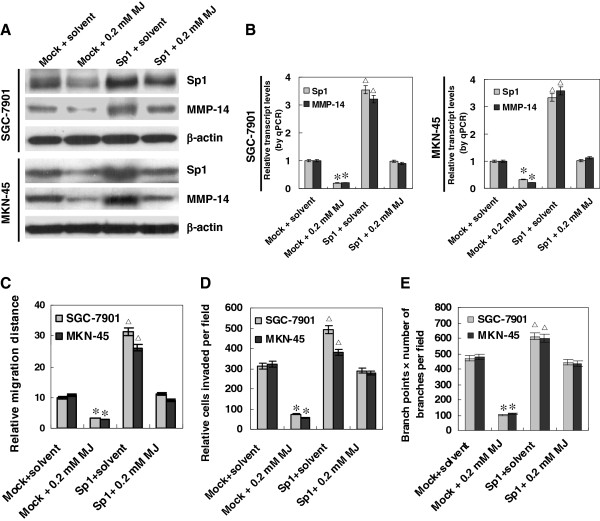
**Restoration of Sp1 rescued sub-cytotoxic MJ-mediated suppression on MMP-14 expression, migration, invasion and angiogenesis of gastric cancer cells.** Human gastric cancer cell lines SGC-7901 and MKN-45 were transfected by Sp1 expression vector for 72 hrs, and incubated with sub-cytotoxic MJ for 24 hrs. **A** and **B**, western blot and real-time quantitative RT-PCR indicated that transfection of SGC-7901 and MKN-45 cells with Sp1 construct rescued the MJ-attenuated expression of Sp1 and MMP-14, when compared to those transfected with empty vector (mock) and treated with solvent. **C**, in scratch migration assay, over-expression of Sp1 promoted the migration of SGC-7901 and MKN-45 cells, and rescued the 0.2 mM MJ-induced inhibition on the migration of cancer cells, when compared to that of solvent-treated mock cells. **D**, transwell analysis indicated that restoration of Sp1 expression rescued the 0.2 mM MJ-induced inhibition on the invasiveness of SGC-7901 and MKN-45 cells, when compared to that of solvent-treated mock cells. **E**, restoration of Sp1 expression in SGC-7901 and MKN-45 cells rescued the 0.2 mM MJ-induced suppression of angiogenesis, when compared to that of solvent-treated mock cells. The symbols (* and △) indicate a significant decrease and a significant increase from solvent-treated mock cells, respectively.

Since above evidence showed that Sp1 participated in the transcriptional regulation of MMP-14 in gastric cancer, we proposed that Sp1 might play an important role in sub-cytotoxic MJ-induced decrease in the migration, invasion and angiogenesis of gastric cancer cells. Human Sp1 expression construct was established and transfected into cancer cells. As shown in Figure 6A and B, transfection of SGC-7901 and MKN-45 cells with Sp1 construct rescued the sub-cytotoxic MJ-attenuated MMP-14 expression. Restoration of Sp1 into SGC-7901 and MKN-45 cell lines rescued the decrease in migration, invasion, and angiogenesis induced by sub-cytotoxic (0.2 mM) MJ (Figure 6C, D, and E). These results suggested that sub-cytotoxic MJ-induced decrease in Sp1 expression contributed to down-regulation of MMP-14 and suppression of migration, invasion and angiogenesis of gastric cancer cells.

## Discussion

In 2002, Fingrut *et al.* first reported the jasmonates-mediated suppression of cellular proliferation and induction of cell death in various human and mouse cancer cell lines, including breast cancer, prostate cancer, melanoma, lymphoblastic leukemia, and lymphoma [6]. In the past decade, several groups have demonstrated that members of jasmonate family and their synthetic derivatives exhibit anti-cancer activity on other kinds of tumor cells, including lung cancer [26], colon cancer [27], glioma [28], cervical cancer [29,30], neuroblastoma [12,13], and myeloid leukemia [31,32]. To date, several mechanisms have been proposed to explain the anti-cancer effects of jasmonates, including induction of severe ATP depletion via mitochondrial perturbation [33], induction of re-differentiation via mitogen-activated protein kinase activity [31], induction of a significant decrease in survivin levels via the β-catenin/T-cell factor pathway [27], and induction of apoptosis via pro-apoptotic proteins of the Bcl-2 family [34], opening the mitochondrial permeability transition pore complex [11] and activation of extrinsic apoptotic pathway [35]. However, the anti-cancer activity of sub-cytotoxic jasmonates and underlying mechanisms still warrant further investigation.

Recent evidence shows that MJ can inhibit melanoma cell migration and suppress the development of melanoma growth in mouse lungs [21], suggesting the potential anti-metastatic activities of MJ. In the current study, we demonstrated that in addition to the cytotoxic properties of MJ in cancer therapy, sub-cytotoxic MJ attenuated the migration and invasion of human gastric cancer SGC-7901 and MKN-45 cells. The SGC-7901 cell line was first established from the metastatic lymph node of a 56-year-old female patient suffering from gastric adenocarcinoma [36], while the MKN-45 cell line was derived from a metastatic liver tumor of a 62-year-old female with gastric cancer [37]. It is well known that the extracellular matrix (ECM) is a barrier to prevent tumor cells from invasion and metastasis [38]. Specific enzymes produced by cancer cells and activated by certain signals, such as matrix metalloproteinases (MMPs), have been reported to degrade ECM, and are associated with the progression of gastric cancer [23,39]. MMP-14, also named as membrane type-1 matrix metalloproteinase, functions as a pericellular collagenase and plays an important role in tumor invasion and metastasis by facilitating the cancer cells to remodel and penetrate ECM [40-42]. Clinical evidence has shown the linkage between high MMP-14 expression and cancer progression, such as lymph node metastases, invasion, poor clinical stage, larger tumor size, and increasing tumor stage [43]. In this study, we found that sub-cytotoxic MJ selectively down-regulated the expression of MMP-14, but not of MMP-7 and MMP-9, in gastric cancer cells. In addition, restoration of MMP-14 rescued the sub-cytotoxic MJ-induced inhibition on the migration and invasion of cancer cells, suggesting the role of MMP-14 down-regulation in the anti-metastatic activities of sub-cytotoxic MJ.

Since MMP-14-mediated degradation of ECM occurs throughout the angiogenic process and contributes to vascular regression [41], we further demonstrated that sub-cytotoxic MJ attenuated the angiogenic capabilities of gastric cancer cells. In addition, sub-cytotoxic MJ did not induce the cell death of human umbilical vein endothelial cells, ruling out the influence of direct cytotoxicity on angiogenesis. In a previous study, MJ was noted to consistently impair the vascular growth in the Chorioallantoic model of angiogenesis [22], while the underlying mechanisms remain largely unknown. VEGF, a dimeric and heparin-binding glycoprotein that functions as a potent mitogen of vascular endothelial cells, is a major inducer of angiogenesis that can promote the growth and metastasis of tumors [25]. In this study, for the first time, we demonstrate that VEGF functions as a downstream gene of MMP-14 in gastric cancer cells. Since our findings indicated that sub-cytotoxic MJ abolished the VEGF expression in gastric cancer cells, combining the evidence that restoration of VEGF expression through MMP-14 over-expression rescued the decrease in tube formation of endothelial cells, we believe that the decreased expression of MMP-14 and downstream VEGF, at least in part, contributes to the anti-angiogenic function of sub-cytotoxic MJ in gastric cancer cells.

To elucidate the mechanisms underlying the down-regulation of MMP-14 by sub-cytotoxic MJ, we further demonstrated that the MMP-14 transcript levels were abolished in MJ-treated gastric cancer cells. Because of the lack of typical TATA box and the presence of a GC-rich sequence immediately upstream of the transcriptional start sites within the promoter, the transcriptional control of MMP-14 is unique compared to other MMP family members [44]. The GC-rich region within human MMP-14 promoter serves as the putative binding site for transcription factor Sp1 in fibrosarcoma and prostate cancer cells [20,44]. In addition, constitutive Sp1 binding to the MMP-14 promoter was also noted in rat glomerular mesangial cells [45]. In this study, we demonstrated the over-expressed Sp1 and its positive correlation with MMP-14 transcription in gastric cancer tissues. Then, we sought to determine whether Sp1 played a role in regulating MMP-14 in gastric cancer cells. Electrophoretic mobility shift assay is a traditional method to investigate the transcription factor-DNA interaction via studying the binding of protein to known DNA oligonucleotide probes [46]. However, this method is an *in vitro* assay and relatively difficult to be quantified [46]. In recent years, ChIP assay is an alternative method to monitor transcriptional regulation through transcription factor-DNA binding interaction in living cells, which is quantitative when coupled with qPCR analysis [46]. Here, we showed that Sp1 bound to the MMP-14 promoter in gastric cancer cells via ChIP assay. Additionally, administration of sub-cytotoxic MJ attenuated the Sp1 expression and binding to MMP-14 promoter, implying the important role of Sp1 in sub-cytotoxic MJ-induced down-regulation of MMP-14 in gastric cancer cells.

## Conclusions

In summary, we demonstrate, for the first time, that sub-cytotoxic MJ attenuates the MMP-14 expression via decreasing the Sp1 expression and binding on MMP-14 promoter, thus inhibiting the migration, invasion and angiogenesis of gastric cancer cells. These findings support the notion that in addition to its cytotoxic activity, sub-cytotoxic MJ exerts anti-metastatic and anti-angiogenic functions as well. These results lay the groundwork for further investigation into the mechanisms of MJ-mediated anti-cancer functions, which is of potential values as a novel therapeutic approach for gastric cancer.

## Competing interests

The authors declare that they have no competing interests.

## Authors’ contributions

QT and LZ conceived and designed study. LZ, DL, and XX performed the experiments. DL provided coordination. MQ, JP, KH, QT, and LZ analyzed the data. QT and LZ provided grant support and wrote the paper. All authors have read and approved the final manuscript.

## Pre-publication history

The pre-publication history for this paper can be accessed here:

http://www.biomedcentral.com/1471-2407/13/74/prepub

## Supplementary Material

Additional file 1: Table S1 Oligonucleotide sets used for constructs and small interfering RNAs. Click here for file

Additional file 2: Table S2 Primer sets used for qRT-PCR. Click here for file

Additional file 3: Figure S1 Time-course effects of sub-cytotoxic MJ on the viability of gastric cancer cells. Human gastric cancer SGC-7901 and MKN-45 cells were incubated with sub-cytotoxic (0.05, 0.1 and 0.2 mM) MJ for 6, 12 and 24 hrs. MTT colorimetric assay indicated that sub-cytotoxic MJ did not affect the viabilities of gastric cancer cells, when compared to those treated by solvent (mock). Click here for file

Additional file 4: Figure S2 Sub-cytotoxic MJ did not affect the proliferation of human endothelial cells. Human endothelial HUVEC cells were incubated with sub-cytotoxic (0.05, 0.1 and 0.2 mM) MJ for 24 hrs. EdU incorporation assay indicated that sub-cytotoxic MJ did not affect the proliferation of HUVEC cells, when compared to those treated by solvent (mock). Click here for file

Additional file 5: Figure S3 Transfection efficiency assay. Confluent monolayers of gastric cancer SGC-7901 and MKN-45 cells were transfected with the enhanced green fluorescent protein (EGFP) reporter vector pEGFP-N1. Seventy-two hrs post-transfection, EGFP expressed within the cytoplasm of cancer cells, with the transfection efficiency around 60%. Click here for file
