# Supplementary material for: Methyl jasmonate abolishes the migration, invasion and angiogenesis of gastric cancer cells through down-regulation of matrix metalloproteinase 14
Source: BMC Cancer. 2013 Feb 10;13:74. doi: 10.1186/1471-2407-13-74 (PMC3576238; doi:10.1186/1471-2407-13-74)
Supplement: Additional file 1: Table S1 — Oligonucleotide sets used for constructs and small interfering RNAs. [file 1471-2407-13-74-S1.pdf]

Supplementary Table S2 Primer sets used for qRT-PCR

Primer set	Primers	Sequence	Product size (bp)
MMP-7	Forward	5'-CAGGCTCAGGACTATCTCAA-3'	278
	Reverse	5'-TCCACTGTAATATGCGGTAA-3'	
MMP-9	Forward	5'-CAGAGATGCGTGGAGAGT-3'	220
	Reverse	5'-TCTTCCGAGTAGTTTTGG-3'	
MMP-14	Forward	5'-GCCTTCTGTTCCTGATAA-3'	225
	Reverse	5'-CCATCCTTCCTCTCGTAG-3'	
VEGF	Forward	5'-ATGACGAGGGCCTGGAGTGT-3'	227
	Reverse	5'-CATTTACACGTCTGCGGATCT-3'	
Sp1	Forward	5'-CTGCCGCTCCCAACTTAC-3'	220
	Reverse	5'-TTGCCTCCACTTCCTCGA-3'	
β-actin	Forward	5'-ATCTACGAGGGGTATGCC-3'	227
	Reverse	5'-TAGCTCTTCTCCAGGGAG-3'	

MMP-7, matrix metalloproteinase 7; MMP-9, matrix metalloproteinase 9; MMP-14, matrix metalloproteinase 14; VEGF, vascular endothelial growth factor; Sp1, specificity protein 1
