# Supplementary material for: Methyl jasmonate abolishes the migration, invasion and angiogenesis of gastric cancer cells through down-regulation of matrix metalloproteinase 14
Source: BMC Cancer. 2013 Feb 10;13:74. doi: 10.1186/1471-2407-13-74 (PMC3576238; doi:10.1186/1471-2407-13-74)
Supplement: Additional file 2: Table S2 — Primer sets used for qRT-PCR. [file 1471-2407-13-74-S2.pdf]

Supplementary Table S1 Oligonucleotide sets used for constructs and small interfering RNAs

RNAs	Sequences
pcDNA3.1-Sp1	5'-CGCCCAAGCTTATGAGCGACCAAGATCAC-3' (sense)<br>5'-CTAGTCTAGATCAGAAGCCATTGCCACT-3' (antisense)
si-Scb	5'-GAACGAUCGAGUAAACGGAtt-3' (sense);<br>5'-UCCGUUUACUCGAUCGUUCtt-3‘ (antisene)
si-MMP14	5'-CCAGAAGCUGAAGGUAGAAtt-3' (sense);<br>5'-UUCUACCUUCAGCUUCUGGtt-3' (antisense)

MMP-14, matrix metalloproteinase 14; Sp1, specificity protein 1; si-Scb, scramble siRNA
