# Supplementary figures and images for: Methyl jasmonate abolishes the migration, invasion and angiogenesis of gastric cancer cells through down-regulation of matrix metalloproteinase 14
Source: BMC Cancer. 2013 Feb 10;13:74. doi: 10.1186/1471-2407-13-74 (PMC3576238; doi:10.1186/1471-2407-13-74)

# Supplementary Figure S1

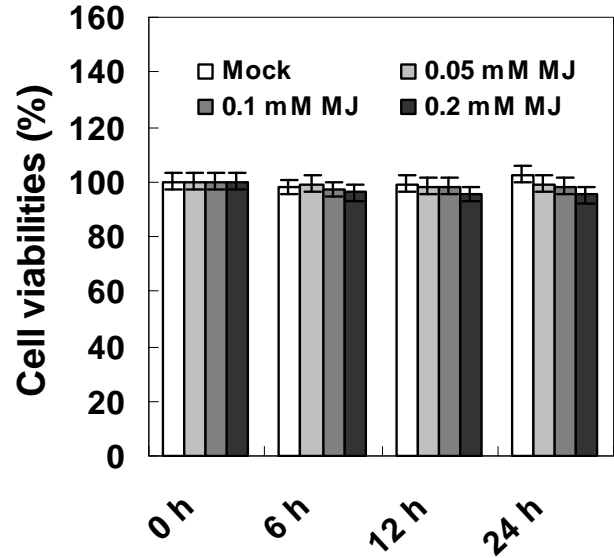

Supplement: Additional file 3: Figure S1 — Time-course effects of sub-cytotoxic MJ on the viability of gastric cancer cells. Human gastric cancer SGC-7901 and MKN-45 cells were incubated with sub-cytotoxic (0.05, 0.1 and 0.2 mM) MJ for 6, 12 and 24 hrs. MTT colorimetric assay indicated that sub-cytotoxic MJ did not affect the viabilities of gastric cancer cells, when compared to those treated by solvent (mock). [file 1471-2407-13-74-S3.pdf]

## Supplementary Figure S2

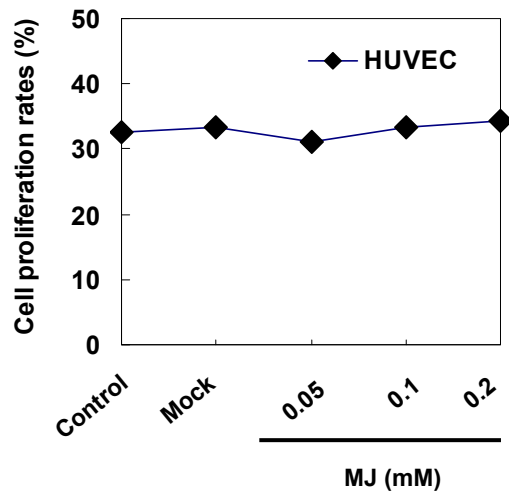

Supplement: Additional file 4: Figure S2 — Sub-cytotoxic MJ did not affect the proliferation of human endothelial cells. Human endothelial HUVEC cells were incubated with sub-cytotoxic (0.05, 0.1 and 0.2 mM) MJ for 24 hrs. EdU incorporation assay indicated that sub-cytotoxic MJ did not affect the proliferation of HUVEC cells, when compared to those treated by solvent (mock). [file 1471-2407-13-74-S4.pdf]

## Supplementary Figure S3

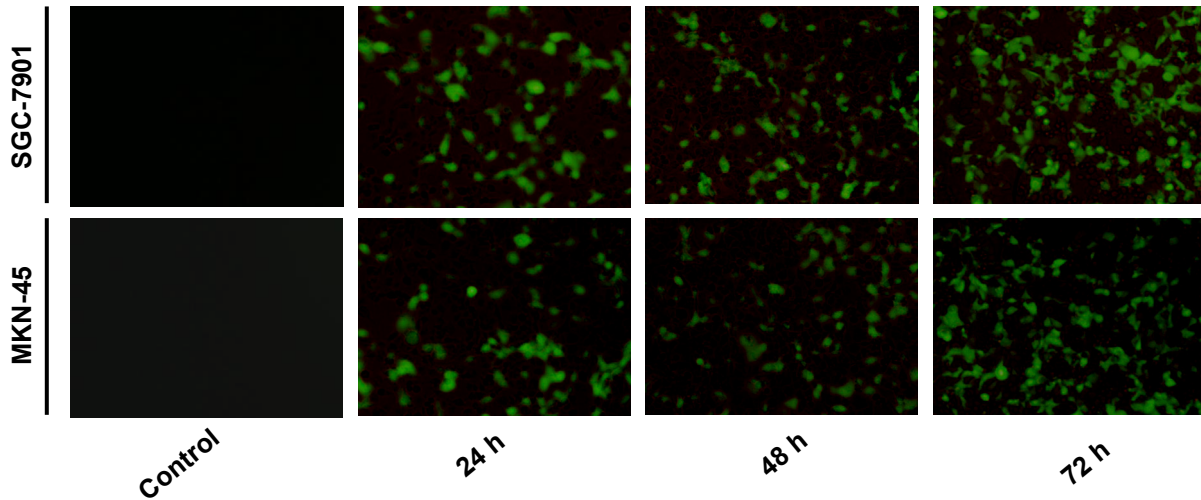

Supplement: Additional file 5: Figure S3 — Transfection efficiency assay. Confluent monolayers of gastric cancer SGC-7901 and MKN-45 cells were transfected with the enhanced green fluorescent protein (EGFP) reporter vector pEGFP-N1. Seventy-two hrs post-transfection, EGFP expressed within the cytoplasm of cancer cells, with the transfection efficiency around 60%. [file 1471-2407-13-74-S5.pdf]
